# The Promotion of Humoral Immune Responses in Humans via SOCS1-Mediated Th2-Bias Following SARS-CoV-2 Vaccination

**DOI:** 10.3390/vaccines11111730

**Published:** 2023-11-20

**Authors:** Xiaoyu Liu, Junyong Han, Renjie Cui, Meifang Peng, Huaidong Song, Rui Li, Gang Chen

**Affiliations:** 1 The Core Laboratory in Medical Center of Clinical Research, Department of Molecular Diagnostic & Endocrinology, Shanghai Ninth People’s Hospital, Shanghai Jiao Tong University (SJTU) School of Medicine, Shanghai 200011, China; xyliubio@163.com (X.L.); rjcui_myyc@163.com (R.C.); pengmeifang816@163.com (M.P.); huaidong_s1966@163.com (H.S.); 2 Fujian Key Laboratory of Medical Measurement, Fujian Academy of Medical Sciences, Fuzhou 350001, China; hanjunyong2002@aliyun.com; 3 Department of Endocrinology, Shanghai Ninth People’s Hospital Affiliated to Shanghai Jiao Tong University School of Medicine, Shanghai 200011, China; 4 Department of Endocrinology, Fujian Provincial Hospital, Fuzhou 350001, China; 5 Shengli Clinical Medical College of Fujian Medical University, Fuzhou 350001, China

**Keywords:** SARS-CoV-2 vaccine, humoral immune responses, single-cell sequencing, Treg cells, the Th1/Th2 balance

## Abstract

The effectiveness of SARS-CoV-2 vaccines varies among individuals. During the COVID-19 global pandemic, SARS-CoV-2 infection showed significant Th1 characteristics, suggesting that the immune disorder and production of SARS-CoV-2 antibodies may be related to Th1/Th2 bias. However, the molecular mechanisms underlying Th1/Th2 bias effects on host immune responses to viruses remain unclear. In this study, the top three subjects with the highest and lowest changes in anti-SARS-CoV-2 antibodies after receiving three doses of SARS-CoV-2 vaccination were selected and defined as the elevated group (E) and the control group (C), respectively. Peripheral blood was collected, single-cell sequencing was performed before and after the third dose of the SARS-CoV-2 vaccine, and the changes in T cell clusters were analyzed. Compared with the C group, the Treg pre-vaccination proportion was lower in E, while the post-vaccination proportion was higher, suggesting that Tregs may be crucial in this process. Differential analysis results of Tregs between the two groups revealed that differentially expressed genes (DEGs) were significantly enriched in the IL4 pathway. Correlation analysis between DEGs and serum antibody showed that the expression of *NR4A2*, *SOCS1*, and *SOCS3* in Tregs was significantly correlated with serum antibodies, suggesting that the immune response in E group changed to Th2 bias, thereby promoting host humoral immune responses. On the other hand, antibody-related genes *SOCS1* and *NR4A2*, as well as lnc-RNA MALAT1 and NEAT1, were highly expressed in the CD4-MALAT1 subclusters. In summary, our study revealed that Th2 bias promotes humoral immune responses in humans by increasing SOCS1 in T cells after SARS-CoV-2 vaccination. Moreover, *NR4A2*, *SOCS1*, MALAT1, and NEAT1 were identified as the potential key biomarkers or treatment targets for enhanced SARS-CoV-2 antibody production by influencing the Th1/Th2 balance in T cells. Our findings have important implications for population stratification and tailored therapeutics for more effective SARS-CoV-2 vaccines.

## 1. Introduction

COVID-19 is a globally prevalent transmitted disease caused by SARS-CoV-2, a variant of coronavirus SARS-CoV which caused the severe respiratory syndrome pandemic in 2002/2003 [1,2]. Recently, three related pandemics have been caused by members of the coronavirus family, including MERS-CoV [2]; thus, novel coronavirus variants or strains that may lead to future pandemics seem inevitable. What is more, SARS viruses are strict human respiratory pathogens, especially in the context of seasonal influenza virus respiratory diseases [3]. SARS-CoV-2 shares similar clinical manifestations, common dissemination mechanisms, infection regions, and seasonal coincidence with the influenza virus. According to clinical reports, influenza virus infection could enhance the ability of SARS-CoV-2 to bind and infect host cells, leading to an increase in COVID-19 infection rate, severe lung damage, and rising mortality [4]. Population studies showed that timely vaccination for SARS-CoV-2 could effectively reduce infection, severe illnesses, and death risks [5,6]. Although the currently approved vaccines have considerable effectiveness, a number of people still cannot generate sufficient antiviral immune responses following vaccination, resulting in low IgG and neutralizing antibody levels [7,8]. Additionally, the continuous emergence of SARS-CoV-2 mutant strains, especially the Delta mutant strain (B.617.2), poses a serious threat to SARS-CoV-2 vaccine effectiveness, leading to a sharp increase in COVID-19 infection rates across many countries [9,10]. Therefore, studies on the mechanisms underlying the differences in human immune response after vaccination will shed insights into virus pathogenesis and the development of effective preventative therapeutics against both SARS-CoV-2 and influenza viruses [11,12,13,14].

As highlighted by the COVID-19 pandemic, the immunophenotypes of SARS-CoV-2 infection display a Th cell skew, producing a significant Th1 profile, and adverse reactions to the virus are the major cause of COVID-19-related morbidity and mortality [15]. A study in Wuhan, China, showed that initial infection with SARS-CoV-2 led to a Th1-dominant response, followed by a Th2-dominant response with disease progression, accompanied by cytokine storms [16]. On the other hand, blood samples from COVID patients showed 100% responses for CD4^+^ T cells and Th1 polarization, with IFN-γ secretion [17]. This suggests that Th1/Th2 bias may play an important role in organ damage with the progression of SARS-CoV-2-induced severe acute respiratory syndrome and possibly the immune responses to SARS-CoV-2 vaccination. A cytokine release caused by Th2 response may be related to antibody production after vaccination. However, it is unclear whether the differences in host humoral immune response after vaccination could be explained by Th1/Th2 bias. Therefore, the mechanisms underlying the different immune responses after coronavirus infection or vaccination need to be further studied.

To study the mechanisms underlying different humoral immune responses after vaccination at the single-cell level, two subject groups were enrolled (three with elevated antibody levels and three controls). Peripheral blood was collected before and after vaccination employing single-cell RNA-seq for the analysis of 12 PBMC samples with 84,331 cells. We found key cell subclusters with significant differences between the two groups, and through differential analysis, candidate regulatory genes and signaling pathways related to humoral responses were identified. Therefore, in this study, we identified the potential molecular regulation mechanisms underlying the different humoral immune responses to COVID-19 vaccination in humans. Our findings provide key insights for personalized therapy against SARS-CoV-2 and future variants.

## 2. Materials and Methods

### 2.1. Clinical Sample Collection and Preparation

Twenty-eight healthy volunteers were initially included in this study, of which eleven received three doses. All subjects were vaccinated with an inactivated SARS-CoV-2 vaccine. Then, the top three subjects with the highest and lowest changes in anti-SARS-CoV-2 antibodies were selected and defined as the elevated group (E) and the control group (C), respectively. The elevated group showed at least a 10-fold increase in post-injection SARS-CoV-2-neutralizing antibody concentration. Peripheral blood samples were collected before and after the third dose of the vaccine. In total, 12 peripheral blood samples (3 pre-injection samples in the E group, 3 post-injection samples in the E group, 3 pre-injection samples in the C group, and 3 post-injection samples in the C group) were sequenced and incorporated in further analyses. Other information, including demographics, disease history, timing of the three-dose vaccination, and serum antibody levels, are summarized in Table S1.

### 2.2. Serum Antibody Concentration Detection

An automated chemiluminescent immunoassay (CLIA) method was utilized to detect SARS-CoV-2 antibody concentrations using AutoLumo A2000 Plus (Zhengzhou Autobio Co., Ltd., Zhengzhou, China). Serum SARS-CoV-2-neutralizing antibody concentration was detected using the SARS-CoV-2 Neutralization Antibody CLIA Microparticles kit (Autobio, CMU0602). Serum SARS-CoV-2 IgG concentration was detected using the SARS-CoV-2 IgG II CLIA Microparticles kit (Autobio, CMU0302). All experiments were performed according to the manufactures’ instructions.

### 2.3. Sample Preparation for Single-Cell Sequencing

Of the fresh peripheral blood, 5 mL was drawn per individual, mixed with 5 mL of phosphate-buffered saline (PBS) and then gently layered over 5 mL of Ficoll (GIBCO, Waltham, MA, USA) in a Falcon tube. The samples were centrifuged at 400 g for 30 min without breaks. Following centrifugation, PBMCs in the white layer were carefully transferred to a new tube. After being washed with PBS twice, the samples were centrifuged at 400 g for 10 min, and the pellets were resuspended in a cryopreservation medium (1 mL of fetal bovine serum supplemented with 120 μL of dimethyl sulfoxide (DMSO)) for long-term storage in liquid nitrogen. Cell viability was assessed via trypan blue staining, and the samples (cell viability > 90%) were prepared using a 10× Genomics Single-Cell 30 v2 Reagent Kit according to the manufacturer’s instructions.

### 2.4. Single-Cell Sequencing and Data Processing

ScRNA-seq was performed on Illumina NovaSeq 6000 Systems using paired-end sequencing (150 nt). The Cell Ranger toolkit (v6.1.1) was utilized to process the raw sequencing data, which were mapped to the GRCh38 human reference genome to generate the gene barcode matrices of the gene counts for each cell. 

The gene barcode matrices were analyzed using the “Seurat” R package (v4.3.0) for quality control [18]. Low-quality cells with the proportion of mitochondrial genes counts at >10% and the number of genes of <500 or >5000 were filtered from downstream bioinformatic analyses. The DoubletFinder R package (v2.0) was utilized to remove potential doublets [19]. Log-Normalize and NormalizeData functions of “Seurat” were used to normalize and log-transform the feature expression of each cell. Then, the normalized expression data of all samples were merged using the merge function into one Seurat object. In order to correct the batch effect introduced by sample merging, mutual matching nearest neighbor (MNN) correction was performed [20]. The gene expression profiles of the top 5000 highly variable genes (HVGs) were determined using the FindVariableFeatures function. Cell clustering was performed using the FindNeighbors() and FindClusters() functions in Seurat. In order to find the optimal clustering resolution, a visualization-based “clustering tree” method was applied [21]. The RunTSNE() and RunUMAP() functions were performed for visualization.

### 2.5. Cell Type Annotations

We performed the unsupervised clustering and differential expression of each cluster to identify the cell types based on the expression of known marker genes from the literature. Eight major cell types were determined with a resolution of 0.3, including CD8^+^ T cells (*CD8A*, *CD8B*), CD4^+^ T cells (*CD4*), natural killer (NK) T cells (*CD8A*, *CD8B*, and *ZNF683*), NK cells (*NKG7*, *FCER1G*, *FGFBP2*, *FCGR3A*, *GZMH*, *TYROBP*, and *IGFBP7*), B cells (*CD74*, *CD79A*, *CD79B*, and *MS4A1*), plasma cells (*JCHAIN*, *MZB1*, and *IGHG1*), monocytes (*LYZ*, *S100A8*, *S100A9*, and *CD14*), and platelets (*PPBP*). After that, T cells and NKT cells were isolated for a repeat round, to identify more refined subclusters with a resolution of 0.7. The processing flow of the second round of clustering was the same as that of the first.

### 2.6. Identification of Signature Genes of the Cell Clusters

Differential expression genes (DEGs) in each cluster were identified employing the FindAllMarkers function in Seurat. The *p*-value of the FDR-corrected Wilcoxon rank sum test was used to determine the levels of significance of these feature genes. Genes that were expressed in more than 10% of the cells, |log2FC| > 0.25, and adjusted *p*-value of < 0.01, were identified as the signature genes.

### 2.7. Quantification and Statistical Analyses

R (version 4.2.3) was used for all statistical analyses. Student’s *t*-test, the Wilcoxon rank-sum test, Pearson’s correlation coefficient, and Spearman’s rank correlation coefficient were utilized. Unless otherwise stated, *p* of <0.05 denoted a significant level.

## 3. Results

### 3.1. Single-Cell Expression Atlas of PBMCs before and after Vaccination

**Figure 1:**
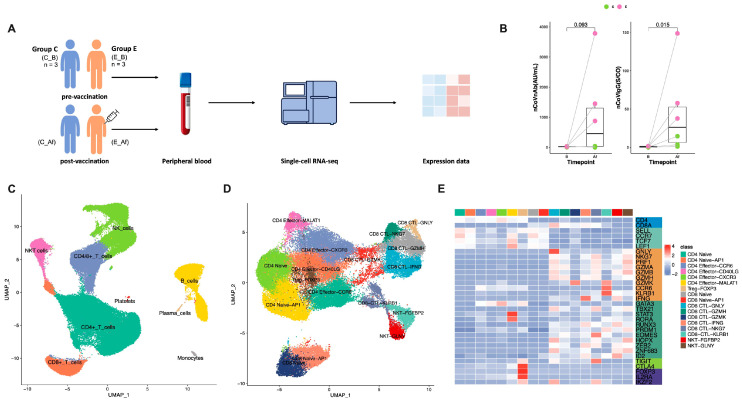
Study design and expression profiling of 84,331 single cells in pre- and post-vaccinated PBMCs. (**A**) Schematic of the study design of the sample composition, processing, and bioinformatic analyses for 12 samples. (**B**) Serum neutralizing antibody and IgG antibody levels in control vs. elevated groups. Median ± IQR; Mann–Whitney U test. (**C**) UMAP plot showing the clusters of all high-quality cells. (**D**) UMAP plot showing clusters of all T cells. (**E**) Heatmap of the marker genes utilized to annotate T cell clusters.

In order to study the mechanisms of differential immune responses to vaccination, the top three subjects with the highest and lowest changes in anti-SARS-CoV-2 antibodies were selected (Table 1 and Figure 1A,B). All six subjects received an inactivated SARS-CoV-2 vaccine, and peripheral blood was collected before and after the third injection of the vaccine. Additional details can be found in Table S1. Deep single-cell RNA sequencing (10X Genomics) was performed on PBMC cells isolated from 12 samples (Figure 1A and Table S1). 

After quality screening, a total of 84,331 single cells with 21,694 expressed genes were incorporated into downstream analysis (Figure 1C). Ten major cell clusters were identified via Uniform Manifold Approximation and Projection (UMAP) clustering [22]. Based on the reported marker genes, the 10 cell clusters were annotated into 8 cell types, including CD8^+^ T cells (*CD8A* and *CD8B*), CD4^+^ T cells (*CD4*), natural killer (NK) T cells (*CD8A*, *CD8B*, and *ZNF683*), NK cells (*NKG7*, *FCER1G*, *FGFBP2*, *FCGR3A*, *GZMH*, *TYROBP*, and *IGFBP7*), B cells (*CD74*, *CD79A*, *CD79B*, and *MS4A1*), plasma cells (*JCHAIN*, *MZB1*, and *IGHG1*), monocytes (*LYZ*, *S100A8*, *S100A9*, and *CD14*), and platelets (*PPBP*) (Figure 1C, Figures S1 and S2). All clusters included cells from multiple patients, indicating that the cells were grouped without patient specificity (Figure S3).

To further address the immune profiles of CD4^+^ and CD8^+^ T cells, NKT cells were clustered into subgroups. Based on the expression patterns of the DEGs, these cells were classified into 17 subclusters (Figure 1D,E). Among them, CD4^+^ T cells were further divided into seven clusters, CD8^+^ cells were divided into eight clusters, and NKT cells were divided into two clusters (Figure 1D,E). Each subgroup showed distinct expression profiles, as depicted by the heatmap analysis of their marker genes (Figure 1E; Table S2). The expression of the signature genes (Figure S4; Table S2) and known functional markers was used to annotate the clusters of CD8^+^ (naive and CTL), CD4^+^ (naive, effector, and Treg), and NKT cells (Figure 1E and Figure S4).

### 3.2. Single-Cell Transcriptional Profiles of T Cells after Vaccination Revealed Differential T Cell Subclusters between Different Humoral Immune Response Groups

**Figure 2:**
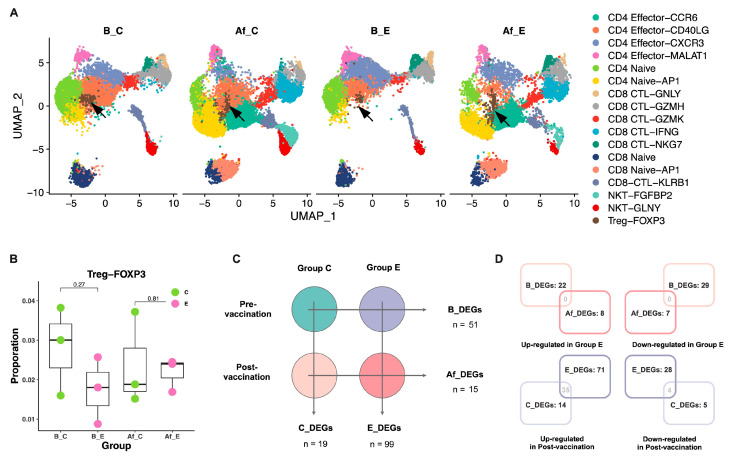
Changes in the Treg cell expression atlas of pre- and post-vaccination were different between the C and E groups. (**A**) UMAP plots showing the clusters of four groups: B_C, Af_C, B_E, and Af_E (arrows indicate Treg cells). (**B**) The ratios of Treg cells in the C vs. E groups. Median ± IQR; Mann–Whitney U test. (**C**) Schematic of the differential analysis design for the four groups. (**D**) Venn plot showing the number of DEGs per group.

To identify the clusters that responded significantly after vaccination, we analyzed the proportion changes in T cell clusters between the two groups. CD4 naive-AP1, CD8 naive-AP1, NKT-FGFBP2, CD4 effector-CCR6, and CD8 CTL-IFNG increased markedly after vaccination, implicit of their important roles in regulating host immune responses following SARS-CoV-2 vaccination (Figure 2A and Figure S5). Contrastingly, we found that the CD4 effector-CCR6, CD4 naive-AP1, and CD8 CTL-IFNG subclusters were increased in both groups, while the CD4 effector-CD40LG, CD4 effector-CXCR3, and CD4 naive subclusters were reduced. Among them, the changes in CD4 naive, CD4 naive-AP1, and CD8 CTL-IFNG were statistically significant (*p* < 0.05) (Figure S5). However, the Treg-Foxp3 subcluster showed different profiles between the two groups, whereby the proportion of Treg cells was lower in the pre-vaccine E group than in the C group and higher in the post-vaccine E group (Figure 2B and Table 2). Regulatory T cells or Treg cells are a subset of CD4^+^ T cells that consistently express high levels of interleukin-2 (IL-2) receptor A chain or CD25, but CD4^+^CD25^+^ cells comprise only 3–10% of the peripheral CD4^+^ T cell population [23]. Regulatory T cells develop in the thymus, characterized by a constitutively high expression of the nuclear transcription factor Foxp3 and cell-surface CD25, and are capable of suppressing excessive immune responses to environmental antigens, microbial antigens, and autoantigens. The differences in Treg cell subsets between the two groups suggested that Treg cells may play an important regulatory role in host humoral immune responses against SARS-CoV-2 vaccination. However, even in terminally differentiated Treg cells, the expression of Foxp3 is not stable. Treg cells may become so-called “exFoxp3” cells or “lapsed Tregs” that acquire a Th1 or Th17 effector memory phenotype after losing Foxp3 expression and regulatory function, a transition that exacerbates the inflammatory response [24]. Th1 polarity affects the morbidity and disease progression of COVID-19, as confirmed by numerous studies.

### 3.3. The Expression Atlas of Treg Cells Was Different between the Two Groups

As visualized by the cluster distributions in the UMAP plot, the expression profile of the Treg cells revealed substantial heterogeneities (Figure 2A). In order to investigate potential reasons for the differences in the Treg cell profiles, we analyzed the differential expression patterns of the two groups before and after vaccination. First, we analyzed the difference between the C and E groups before and after vaccination (Figure 2C). The results showed that there were more DEGs before vaccination than after vaccination (51 vs. 15), indicating that the Treg cell status of the two groups was significantly different before vaccination, and the body mobilized similar regulatory pathways in response to vaccination (Figure 2D and Figure S6A–D). The pathway enrichment of DEGs showed that the PI3K–AKT signaling pathway, related to cell proliferation, was inhibited in the Treg cells of the E group before vaccination (B_E group) (Figure S6E,F). This result may explain the lower proportion of Treg cells in group B_E than that in group B_C.

**Figure 3:**
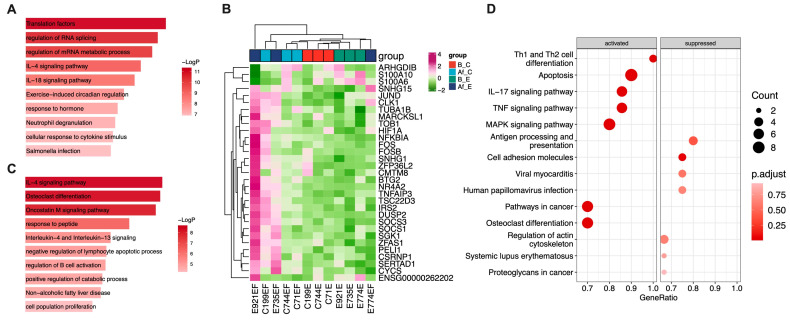
Enrichment pathway analysis of significantly changed genes. (**A**) Top 10 enriched pathways of the DEGs in the E group (n = 99) via Metascape (https://metascape.org) (accessed on 7 September 2023). (**B**) Heatmap of the significant antibody-related genes (n = 31). Hierarchical clustering identifies four groups. (**C**) Top 10 enriched pathways of the 31 antibody-related genes via Metascape. (**D**) Top enriched KEGG pathways of the 31 antibody-related genes via “clusterProfiler” R package.

Then, we analyzed the difference between group B_E vs. Af_E and group B_C vs. Af_C (Figure 2C,D). More DEGs were found in group E than in group C (99 vs. 19), suggesting that the Treg cells in E underwent greater differentiation and activation after vaccine stimulation. Pathway enrichment showed that the DEGs of group E were significantly enriched in the IL-4 signaling pathway (28 of 99 DEGs were enriched) (Figure 3A) [25], indicating that changes in the Treg cell expression profiles in group E may be related to Th2 cell differentiation.

### 3.4. The DEGs of the Treg Cells Correlated with Humoral Immune Responses

To study the relationship between the DEGs and serum antibody levels, a correlation analysis of all DEGs (146 in total) and serum antibody levels was performed (Table S3). The results showed that a total of 31 DEGs were significantly correlated with serum neutralizing antibody (nAbs) or IgG antibody levels after SARS-CoV-2 vaccination (|r| > 0.5, *p* < 0.05; Figure 3B and Table S3). On the other hand, the expression changes in the 41 DEGs were significantly associated with the difference in serum antibody levels before and after vaccination (|r| > 0.5, *p* < 0.05), as shown in Table S4. The pathway enrichment analysis of the 31 DEGs showed that most antibody-related DEGs (11/31) were enriched in the IL-4 signaling pathway (Figure 3C) [25]. At the same time, KEGG enrichment analysis showed that the pathways of Th1 and Th2 cell differentiation were activated after vaccination (Figure 3D), suggesting that host humoral immune responses after vaccination may be related to the Th1/Th2 balance. 

Among 31 Ab-related genes, *SOCS1*, *SOCS3*, and *NR4A2* were reported to be related to the stability of Treg cells and regulation of the Th1/Th2 balance [26,27,28]. There was a significant positive correlation between the expressions of these genes and serum antibody levels. 

### 3.5. The CD4^+^ Cell Subcluster with High Expression of LncRNA MALAT1 and NEAT1 Was Associated with Enhanced Humoral Immune Responses

**Figure 4:**
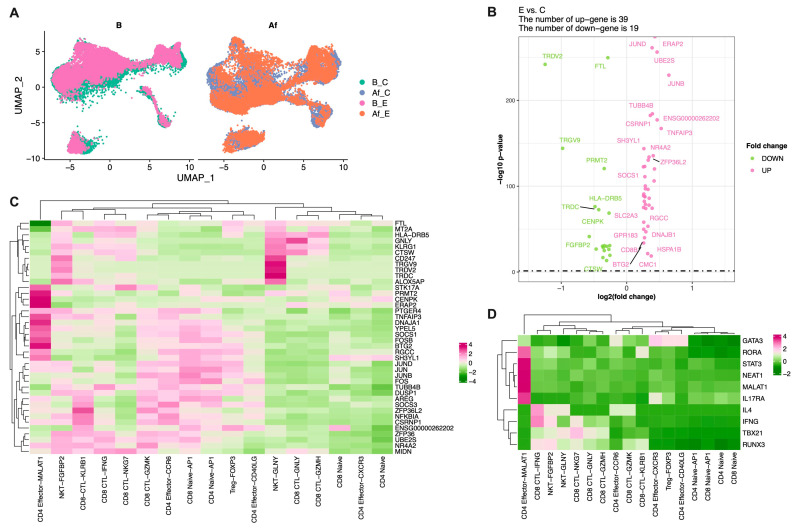
Differential expression atlas of CD4 effector T cells was correlated with host humoral immune responses. (**A**) UMAP plots of four groups with no clustering: B_C, Af_C, B_E, and Af_E. (**B**) Volcano plots showing the increase in CD4 differentiation genes *NR4A2*, *SOCS1*, and *SOCS3* in Af_E cells compared with in Af_C. (**C**) Heatmaps of the significantly expressed antibody-related genes (n = 41) in all T cell clusters. (**D**) Heatmaps of marker genes and key transcription factors.

To investigate the differential expression profiles of the two groups after vaccination, differential analysis was performed (with no clustering) for all Af cells (Figure 4A). A total of 58 DEGs were found to be significantly different between the E and C groups (Figure 4B; Table S5). In order to study whether the differential expression profiles were related to serum antibody levels, correlation analysis were further carried out between the expression values of the 58 DEGs and serum antibody levels. A total of 41 DEGs were significantly correlated with the serum neutralizing antibody or IgG antibody levels (|r| > 0.5), as shown in Figure 4C and Table S6. Ab-related genes were highly expressed in the CD4-MALAT1 subcluster, indicating the important roles of these cells in regulating host immune response after vaccination. Notably, the ab-related gene *SOCS1* was significantly highly expressed in this subcluster, and the expression of *NR4A2* was also relatively high (Figure 4C).

Based on the marker genes of the CD4-MALAT1 subcluster, we found that these cells were highly expressed lncRNA *MALAT1* and *NEAT1* (Figure 4D and Figure S7A; Table S2). Several studies on multiple sclerosis and asthma found that MALAT1 and NEAT1 regulate the RUNX3–GATA3/TBX21 axis, key transcription factors (TFs) in T cell differentiation, by regulating the expression levels of the related miRs, thereby influencing the direction of the Th1/Th2 bias. What is more, CD4-MALAT1 possessed classical Th17 phenotypes, with the high-expression of *STAT3*, *RORA*, and *IL17RA* (Figure 4D and Figure S2). As shown in Figure S7B, the Th17 cell differentiation pathway (hsa04659) was significantly changed. *TBX21*, a key transcription factor (TF) of Th1 differentiation, was downregulated in this cluster, whereas *RUNX1* was upregulated. Retinoic acid receptor-related orphan nuclear receptor γ (RORγ) is a key TF that induces Th17 differentiation, and its expression is influenced by environmental factors, thus having substantial functional plasticity [29]. When the inflammatory environment changes, Th17 cells tend to acquire Th2-related cytokine expression profiles [29,30]. In studies on allergic asthma, Th17 polarization was found during the acute stage of asthma; however, this phenotype was subsequently transformed into a more pathogenic Th2 phenotype with IL-17-producing ability, maintaining persistent mixed eosinophilic and neutrophilic inflammation during the chronic stage of asthma [31]. These findings further suggest the vital influence of Th2 bias during the host immune response process following SARS-CoV-2 vaccination. 

## 4. Discussion

**Figure 5:**
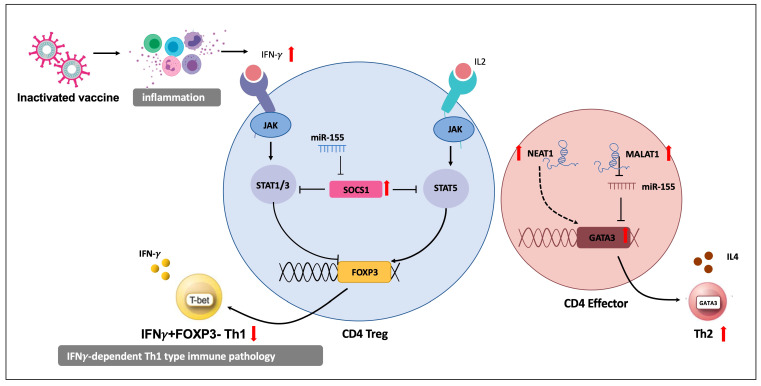
Schematic diagram of the differential immune response mechanism. The up and down arrows indicate increases and decreases, respectively.

Vaccines are considered the most effective means of human protection against viral infections. Studies have shown that timely SARS-CoV-2 vaccination could effectively reduce the risk of infection, severe disease, and death [5,6]. Nevertheless, considerable variations in vaccine effectiveness among populations still remain. In this study, we carried out scRNA-seq analysis to investigate the single-cell expression profiles of individual differential responses after vaccination, along with their possible underlying molecular mechanisms (Figure 5). The findings highlight the potential molecular regulation mechanisms underlying differential humoral immune responses to COVID-19 vaccination in humans, providing key insights into personalized therapy against SARS-CoV-2 and future variants.

We found that the changes in Treg cells in people with elevated antibody levels were significantly different from those in the control group after vaccination. Several studies have shown that Tregs may acquire a Th1 or Th17 effector memory phenotype after losing Foxp3 expression, a process that may exacerbate the degree of inflammatory response [24]. The expression of Foxp3 is elevated by the activation of some TFs, including nuclear factor–kappa B (NF-kB), nuclear factor of activated T cells (NFAT), and Smad3 [28,32,33,34,35]. In this study, the genes related to the regulation of Treg cell stability, SOCS1, SOCS3, and NR4A2, were highly expressed in group E, and DEGs were significantly enriched in the IL4 pathway. Since IL-4 is a key Th2-promoting cytokine, our results suggest a Th2-oriented response in group E, resulting in enhanced humoral immune responses.

*SOCS1* and *SOCS3* belong to the suppressors of the cytokine signaling (SOCS) family that can negatively regulate the JAK–STAT pathway. It was found that *SOCS1* and *SOCS3* play important roles in Th cell differentiation [36]. SOCS1 controls the homeostasis of nTregs by regulating IL-2-mediated STAT5 signaling [37,38]. It negatively regulates Treg number but is necessary for the function of Tregs. It was reported that the number of Foxp3^+^ CD4 T cells increased in the thymus and spleen of Treg-specific SOCS1-knockout mice [26]. Despite the increased number of nTregs, SOCS1-deficient mice developed IFN-γ-dependent Th1 type immunopathology [27]. In addition, miR155, a key regulator of *SOCS1*, has been found to be crucial in maintaining Treg homeostasis [38]. The deletion of miR155 significantly increased the expression of SOCS1 in Tregs, resulting in a decreased sensitivity of Treg to IL-2 and the loss of FOXP3 expression [39]. Therefore, *SOCS1* plays a vital role in protecting Tregs from conversion into effector T cells under the harmful influence of inflammatory cytokines [40]. However, the role of *SOCS3* in Tregs is still unclear. It is worth noting that SOCS3 could inhibit Th1 immune responses through the IL-12-mediated activation of STAT4 [36]. Alongside that, SARS-CoV-2 and influenza viruses have been reported to induce non-specific intrinsic virulence systems, including SOCS, as checkpoint inhibitors [41,42,43,44]. The vital role of vitamin D and microbiota in immune responses, including autoimmunity, *SOCS1*, and SOCS3, have been reported to be involved in Vitamin D metabolism through the VDR-miRNA155-SOCS1 pathway, negatively regulating inflammation and disease pathogenesis [45,46,47,48,49,50]. In addition, *SOCS1* may also affect the differentiation and function of Th17 through miRNA155 in asthma and systemic sclerosis [51,52]. *SOCS3* was also shown to play a role in modulating Th17 differentiation, as well as contributing to the progression of osteoarthritis and acantholysis [53,54]. Moreover, some studies have shown SOCS1 and SOCS3 as essential mediators of immune tolerance in inflammatory diseases by maintaining microbiota homeostasis [55,56,57,58]. *NR4A2* (Nurr1), a member of the orphan nuclear receptor 4A (NR4As) family, was originally cloned as an immediate–early response gene regulated by growth factors, with a crucial role in the control of cell proliferation, differentiation, and survival [32]. NR4A2 can bind to Foxp3 regulatory regions and control CD4^+^ T cell function by inducing Foxp3^+^ Treg cell generation and inhibiting cytokine production by effector T cells. It has been reported that NR4A2-deficient T cells inhibit Foxp3 expression, leading to an abnormal induction of Th1 cells and aggravation of colitis [28]. Nevertheless, apart from its role in modulating Treg cell function, NR4A2 has also been shown to regulate vitamin D metabolism, Th17 cell differentiation, and microbiota homeostasis in some inflammatory autoimmune diseases, such as inflammatory bowel disease and Graves’ diseases [59,60,61,62]. Thus, given the important regulatory roles of vitamin D, Th17, and the microbiota in inflammatory diseases, there may be other possible mechanisms underlying the observed modulatory effects of SOCS1/SOCS3 and NR4A2 on differential vaccination responses in this study, which deserve future study.

The expression profiles of the CD4-MALAT1 subcluster were significantly different between the E and C groups after vaccination, and these differences were significantly associated with serum antibody levels, suggesting its important role in host immune response against SARS-CoV-2. Notably, the ab-related gene *SOCS1* was significantly highly expressed in this subcluster, and *NR4A2* expression was also relatively high (Figure 4C). The CD4-MALAT1 subcluster, specifically highly expressed LncRNAs MALAT1 and NEAT1. MALAT1 (metastasis-associated lung adenocarcinoma transcript 1) also known as NEAT2, is an LncRNA first reported in 2003 [63]. MALAT1 is widely expressed in mammalian normal tissues and abnormally expressed in many human malignant tumors, which alters the biological phenotype of tumor cells [64]. NEAT1 (Nuclear Enriched Abundant Transcript 1) is considered as an oncogene in solid tumors such as prostate cancer, hepatocellular carcinoma, gastric cancer, colorectal cancer, and glioma, which promotes the proliferation, metastasis, and drug resistance of tumor cells [65]. Some studies in multiple sclerosis and asthma showed that lncRNA MALAT1 directly downregulates miR-155 miRNA [66,67]. MALAT1 and NEAT1 regulate RUNX3–GATA3/TBX21 levels, a key axis of the transcription factor during T cell differentiation, by regulating the expression of the related miRNA [68]. Thus, Th1/Th2 differentiation is influenced, which consequently leads to an IFNγ/IL4 imbalance. Consistent with the above findings, our study further showed the important effect of the Th1/Th2 balance in host humoral immune responses after SARS-CoV-2 vaccination. What is more, the CD4-MALAT1 subcluster presented Th17 and naïve phenotypes (high expression of *CCR7*, *TCF7*, and *LEF1*). The function of the mutual cross-talks of Th1, Th2 and Th17 in the immune response process during inflammation or vaccination deserves future studies.

Finally, we proposed a potential mechanism for differential humoral immune response after COVID-19 vaccination (Figure 5). Inactivated SARS-CoV-2 stimulates host interferon response, with the production of large amounts of interferons in the body [69,70,71]. IFNγ-induced STAT1 can disrupt Foxp3 expression, inducing the conversion of nTregs into Th1 cells. In individuals with high antibody levels, an enhanced expression of SOCS1 maintains Treg function by suppressing the IFNγ signaling pathway. Alongside that, the high expression of LncRNAs MALAT1 and NEAT1 serve as key regulators that promote Th2 phenotype transformation. MALAT1 could directly downregulate miR-155 miRNA. Therefore, MALAT1-mediated miR-155 deletion may decrease T-bet protein levels, while increasing GATA-3 protein levels, promoting Th2 cytokine production (IL-4, -5, and -13). Hence, the enhanced expression of *SOCS1* and *MALAT1* in the E group may play a crucial role in suppressing Th1 differentiation while facilitating the Th2 phenotype with an enhanced humoral immune state.

It is also noteworthy that this mechanism highlights the important role of miR-155 in the immune response against SARS-CoV-2. MiR-155 is a well-known inflammation-related miRNA that influences the immune system in various diseases [72,73]. MiR-155 represents a major regulator of inflammation during disease progression, including COVID-19 [74,75]. It has been found that miR-155 is relatively upregulated in the PBMCs of COVID-19 patients and negatively correlated with SOCS-1 expression. MiR-155 was also suggested as a valuable diagnostic and prognostic factor in COVID-19 [39]. However, the exact effects of miR-155, especially its role in host immune responses in COVID-19, need to be confirmed by further studies. 

Some limitations of the study merit discussion. First, the sample size and number of cells in this study were limited, and further expansion of the study cohort is needed to better elucidate the population immune diversity. Second, our study only conducted the scRNA-seq to analyze the expression profile; there was a lack of methods such as ImmuneRepertoire sequencing (IR-SEQ) that specifically shows the diversity of B and T lymphocytes. ScRNA-seq-integrated IR-SEQ analysis can help us better understand the specific clonotypes of post-vaccine responses. Finally, our study used a range of bioinformatics tools in an attempt to explain the molecular mechanisms underlying the differences in response to vaccines. However, further in vivo and in vitro experiments are needed to validate the causality of the SOCS1-mediated Th cell differentiation and the difference in antibody production.

In summary, our study showed a comprehensive landscape of differential cell expression before and after SARS-CoV-2 vaccination and revealed that Th2 bias promotes humoral immune responses in humans by increasing SOCS1 in T cells after SARS-CoV-2 vaccination. Our study also identified the potential key biomarkers or treatment targets for enhanced SARS-CoV-2 antibody production, that may act by influencing the Th1/Th2 balance in T cells. In addition, our findings have important implications for population stratification and tailored therapeutics for more effective SARS-CoV-2 vaccines, providing further insights into the mechanisms underlying differential host immune responses to COVID-19 infection.

## Figures and Tables

**Table 1:** Serum neutralizing antibody (nAbs) and IgG antibody levels of six subjects.

		Pre-Vaccination		Post-Vaccination	
ID	Group	nCoVnAbs (AU/mL)	nCoVIgG (S/CO)	nCoVnAbs (AU/mL)	nCoVIgG (S/CO)
C71	C	5.095	0.080	40.646	1.091
C744	C	36.01	0.158	0.906	14.526
C199	C	9.52	0.034	30.924	3.565
E735	E	22.764	1.661	1450.328	57.69
E921	E	23.956	1.536	3782.458	148.56
E774	E	30.283	1.343	875.019	37.705

**Table 2:** Tregs proportions in the E and C groups (pre- and post-vaccination).

Sample	Group	Ratio—Pre (%)	Mean—Pre (%)	Ratio—Post (%)	Mean—Post (%)
C199E	C	0.038		0.015	
C71E	C	0.030		0.037	
C744E	C	0.016	0.028	0.019	0.024
E735E	E	0.026		0.024	
E774E	E	0.009		0.017	
E921E	E	0.018	0.018	0.024	0.022

## Data Availability

The processed scRNA-seq data were deposited in the GEO database under accession number GSE244173. The raw scRNA-seq reads were deposited in the Genome Sequence Archive (GSA) under accession number HRA005764. A request for access to the raw data can be made by completing an application form through the GSA–Human System.

## Supplementary Materials

The following supporting information can be downloaded at: https://www.mdpi.com/article/10.3390/vaccines11111730/s1, Figure S1: Expression levels of the marker genes for eight types of cells, including CD8^+^ T cells (*CD8A* and *CD8B*), CD4^+^ T cells (*CD4*), natural killer (NK) T cells (*CD8A*, *CD8B*, and *ZNF683*), NK cells (*NKG7*, *FCER1G*, *FGFBP2*, *FCGR3A*, *GZMH*, *TYROBP*, and *IGFBP7*), B cells (*CD74*, *CD79A*, *CD79B*, and *MS4A1*), plasma cells (*JCHAIN*, *MZB1*, and *IGHG1*), monocytes (*LYZ*, *S100A8*, *S100A9*, and *CD14*), and platelets (*PPBP*); Figure S2: Heatmap of the marker genes utilized to annotate eight cell clusters; Figure S3: Composition of the cell types. (A) Number and proportion of each major cell cluster in the 12 samples. (B) Number and proportion of each T cell subcluster in the 12 samples; Figure S4: Expression levels of the marker genes for 17 types of T cells; Figure S5: Boxplots of 17 T cell subcluster ratios in the C vs. E groups. Median ± IQR; Mann–Whitney U test; Figure S6: Treg cell expression atlas pre- and post-vaccination changes were different between the C and E groups. (A) Volcano plots showing the DEGs of B_E vs. B_C Tregs. (B) Volcano plots showing the DEGs of Af_E vs. Af_C Tregs. (C) Volcano plots showing the DEGs of Af_C vs. B_C Tregs. (D) Volcano plots showing the DEGs of Af_E vs. B_E Tregs. (E) Top enriched KEGG pathways of the 51 B_E DEGs analyzed using the “clusterProfiler” R package. (F) Visualized PI3K-AKT signaling pathway of the expression profile data of B_E Tregs; Figure S7: *STAT3*, *RORA*, and *IL17RA* were highly expressed in CD4-MALAT1 T cells. (A) Expression levels of the marker genes of CD4-MALAT1 T cells. (B) Visualized Th17 cell differentiation pathway of the expression profile data of CD4-MALAT1 T cells; Table S1. Detailed information of all subjects; Table S2. The expression of top 50 signature genes and known functional markers of all T subclusters; Table S3. Correlation analysis of all DEGs and serum antibody level; Table S4. Correlation analysis of the expression changes of DEGs and the difference of serum antibody levels pre- and post-vaccination; Table S5. Differential analysis (with no clustering) of the two groups; Table S6. Correlation analysis of all DEGs and serum antibody level.
