# Supplementary figures and images for: The Promotion of Humoral Immune Responses in Humans via SOCS1-Mediated Th2-Bias Following SARS-CoV-2 Vaccination
Source: Vaccines (Basel). 2023 Nov 20;11(11):1730. doi: 10.3390/vaccines11111730 (PMC10674672; doi:10.3390/vaccines11111730)

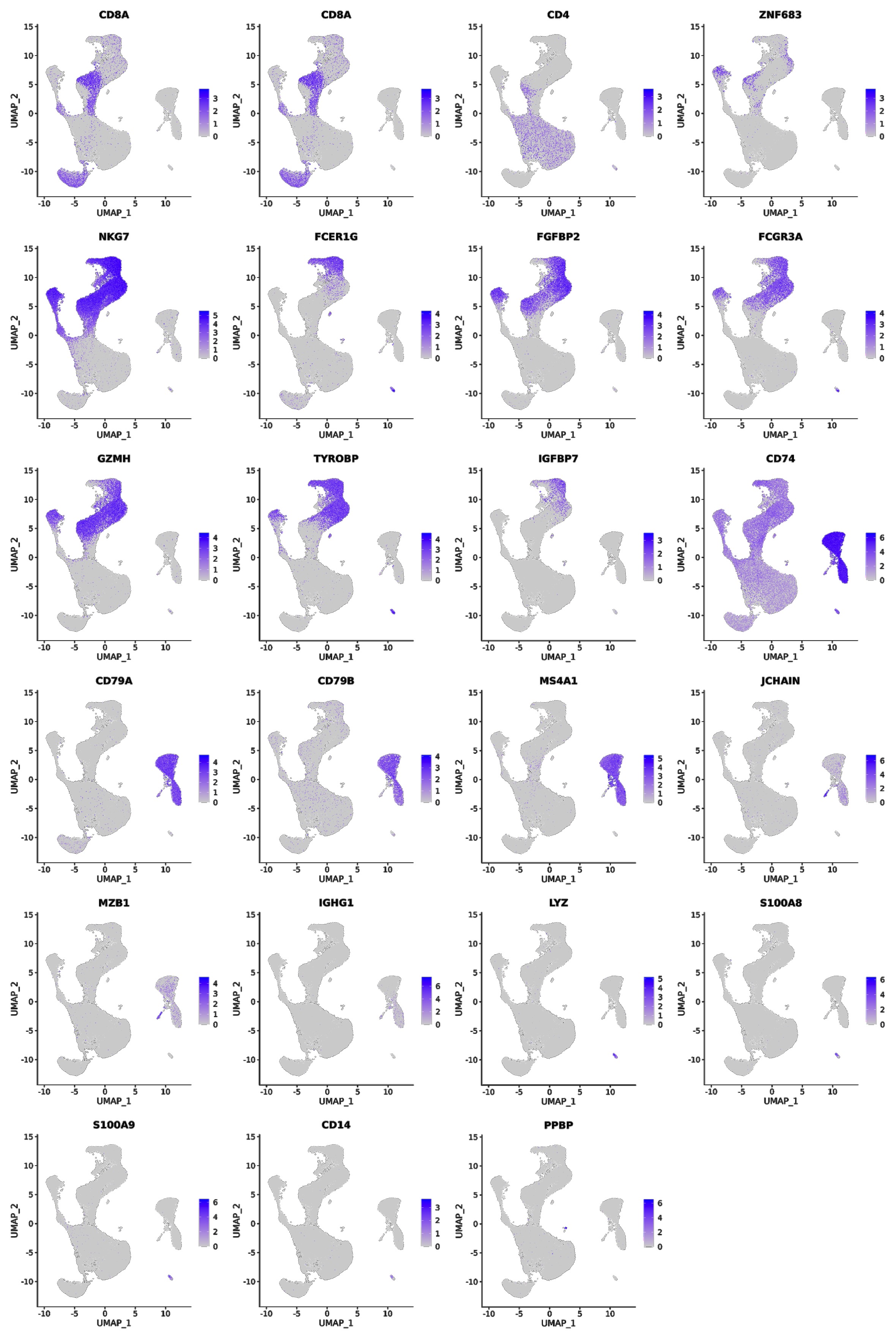

Supplement: Supplementary file 1 [file vaccines-11-01730-s001.zip › vaccines-2662602-supplementary/Supplementary Files/FigS1.jpg]

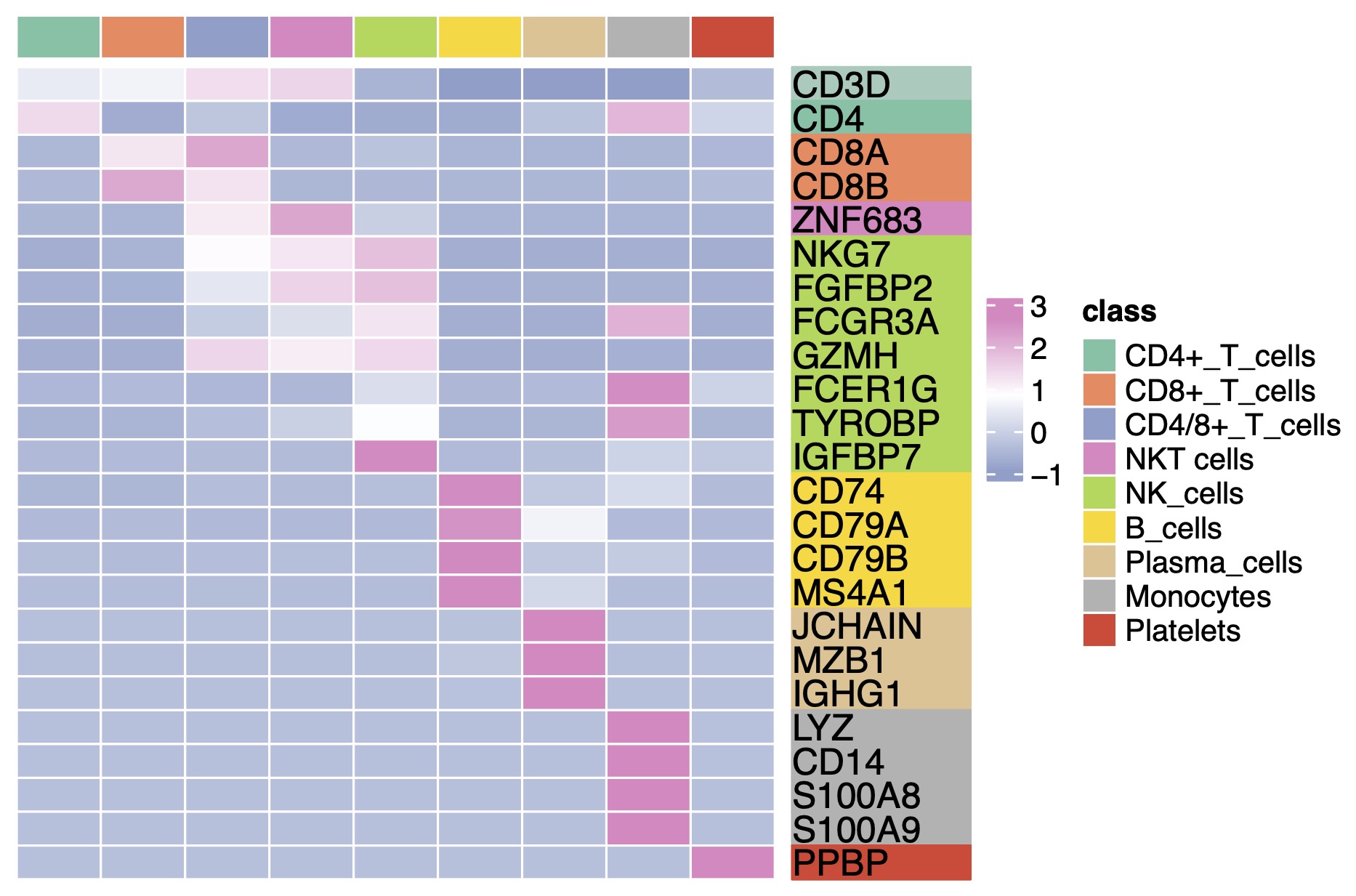

Supplement: Supplementary file 1 [file vaccines-11-01730-s001.zip › vaccines-2662602-supplementary/Supplementary Files/FigS2.jpg]

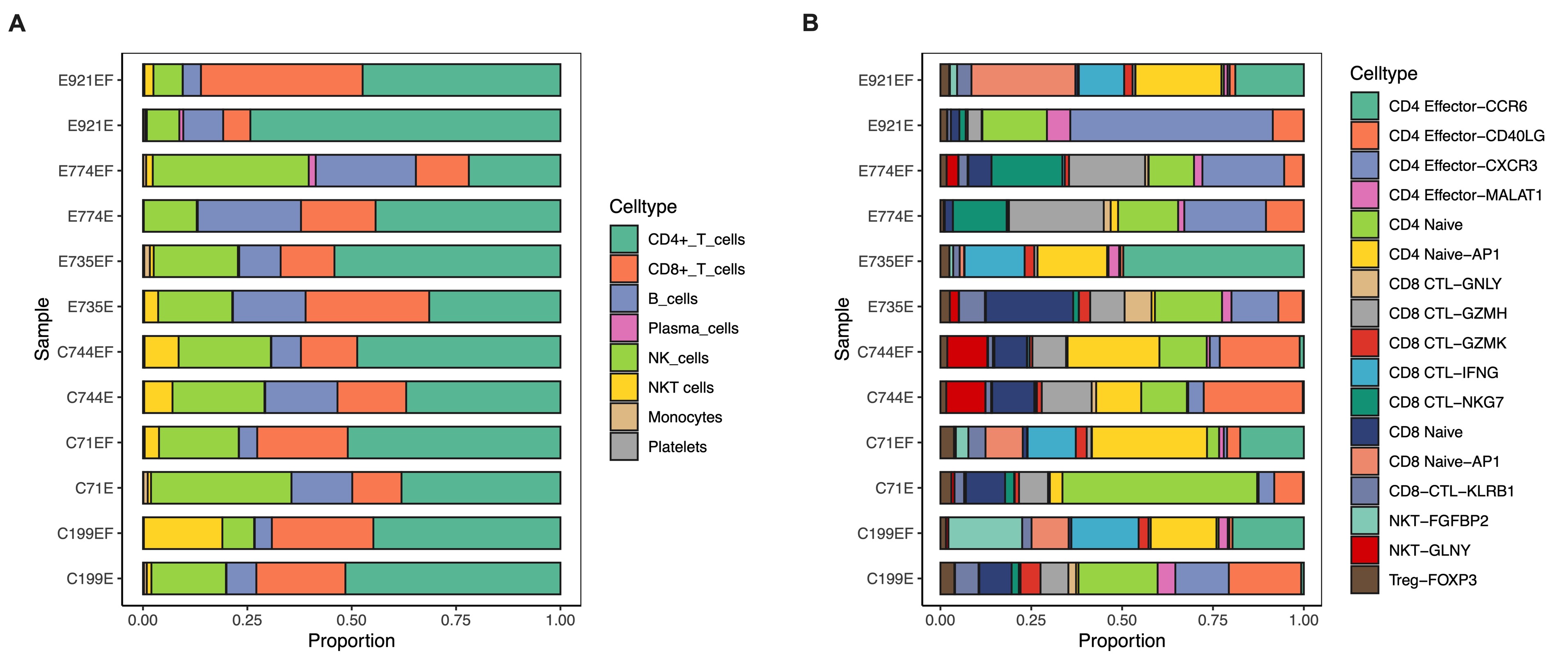

Supplement: Supplementary file 1 [file vaccines-11-01730-s001.zip › vaccines-2662602-supplementary/Supplementary Files/FigS3.jpg]

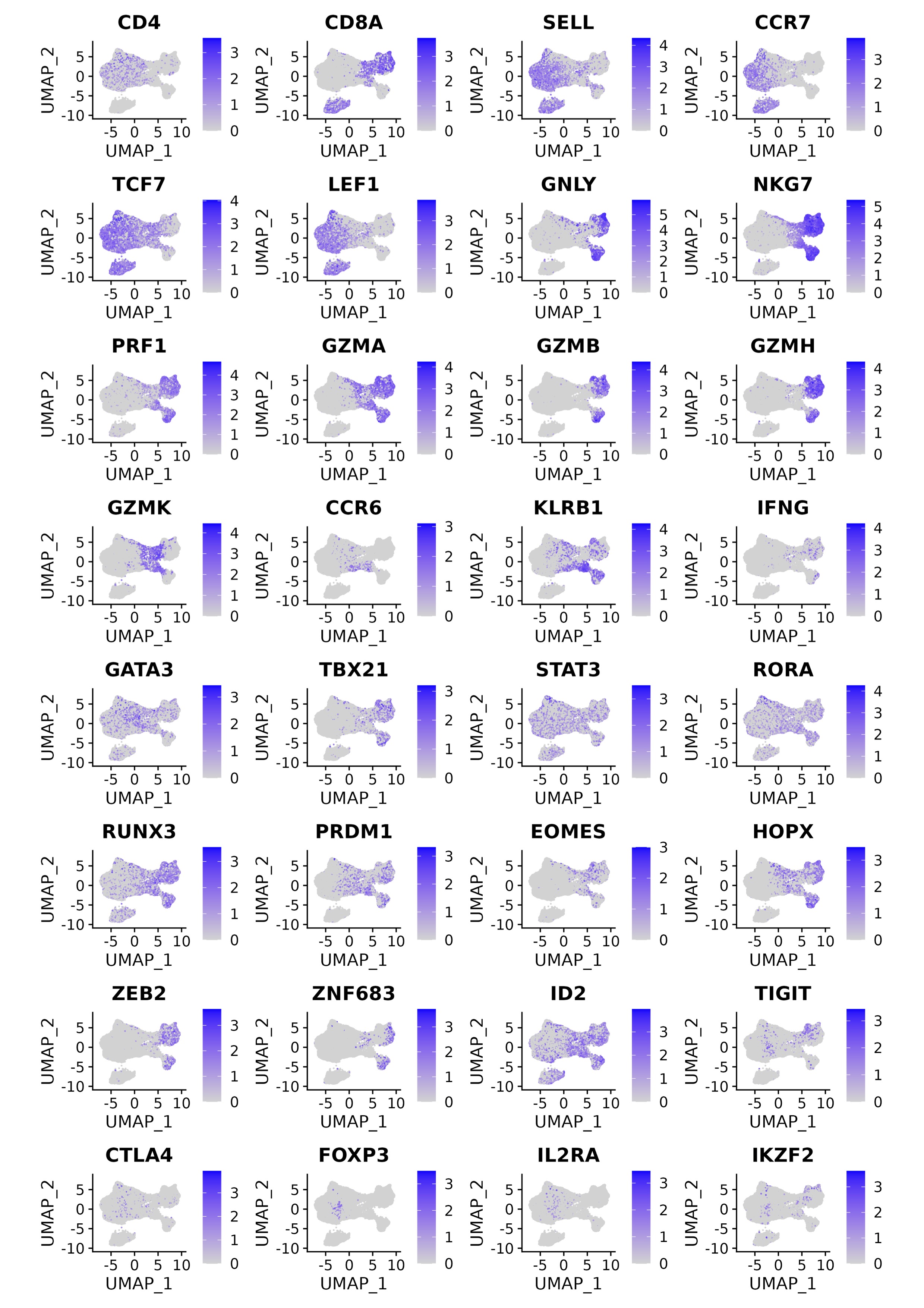

Supplement: Supplementary file 1 [file vaccines-11-01730-s001.zip › vaccines-2662602-supplementary/Supplementary Files/FigS4.jpg]

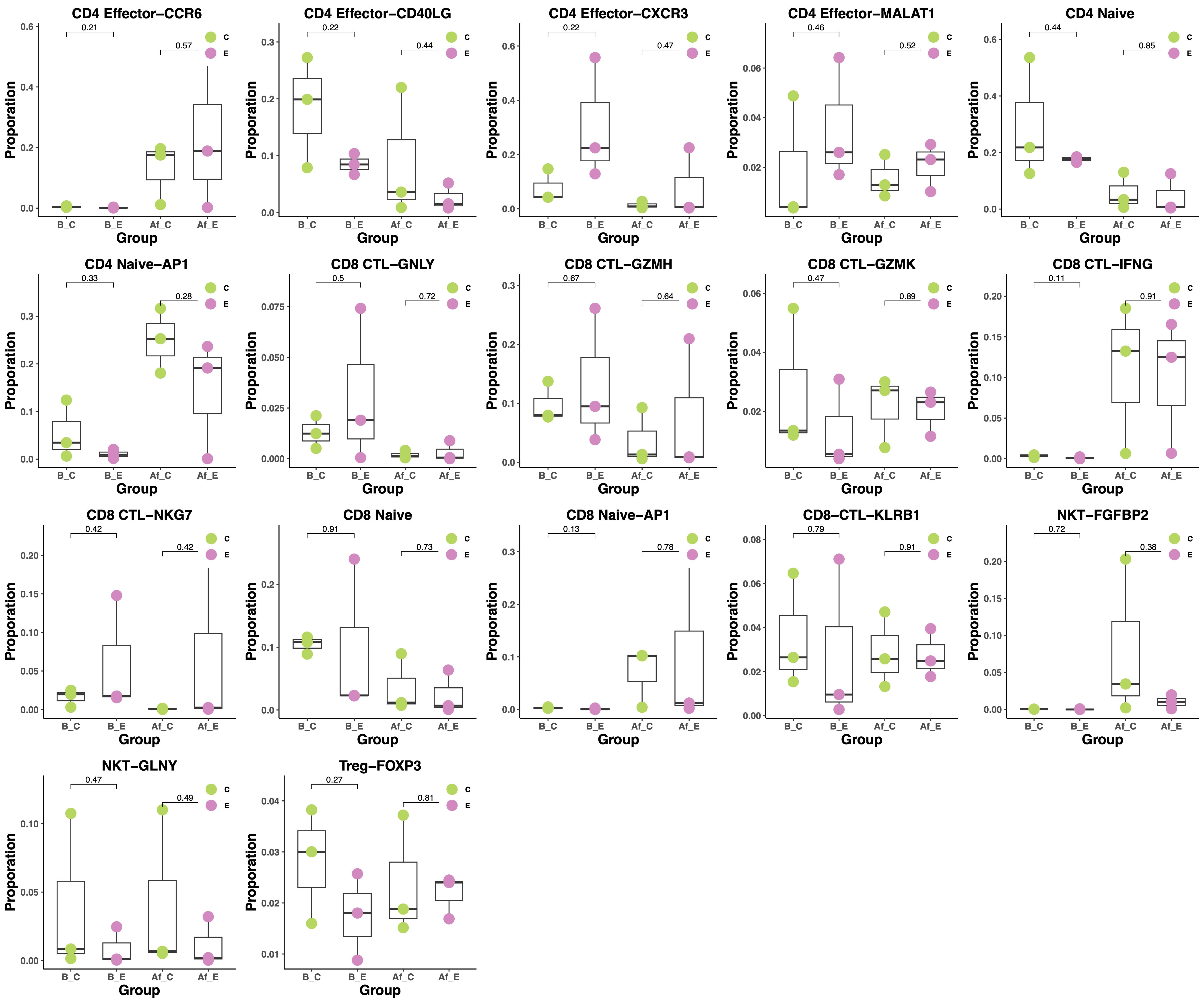

Supplement: Supplementary file 1 [file vaccines-11-01730-s001.zip › vaccines-2662602-supplementary/Supplementary Files/FigS5.jpg]

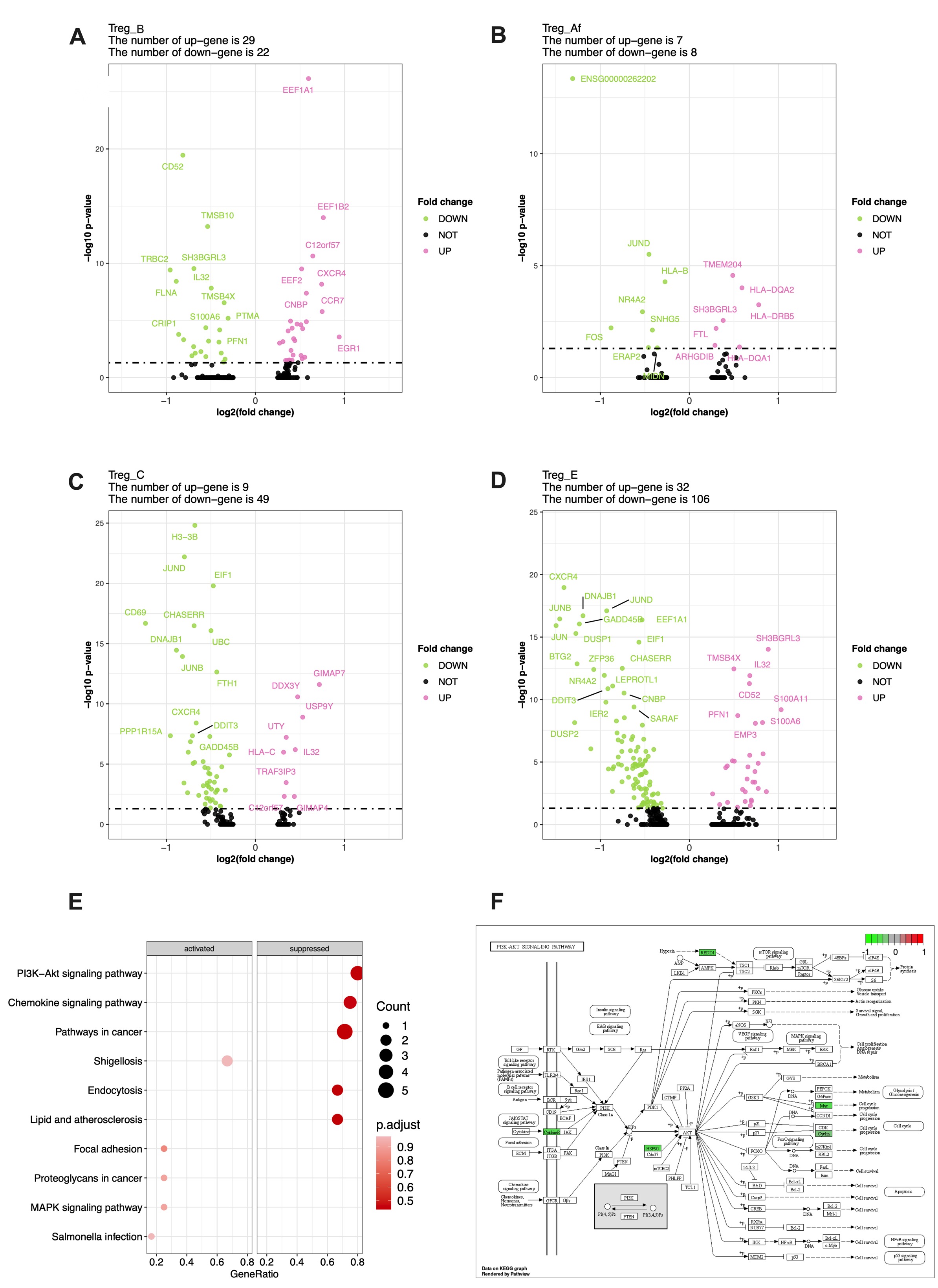

Supplement: Supplementary file 1 [file vaccines-11-01730-s001.zip › vaccines-2662602-supplementary/Supplementary Files/FigS6.jpg]

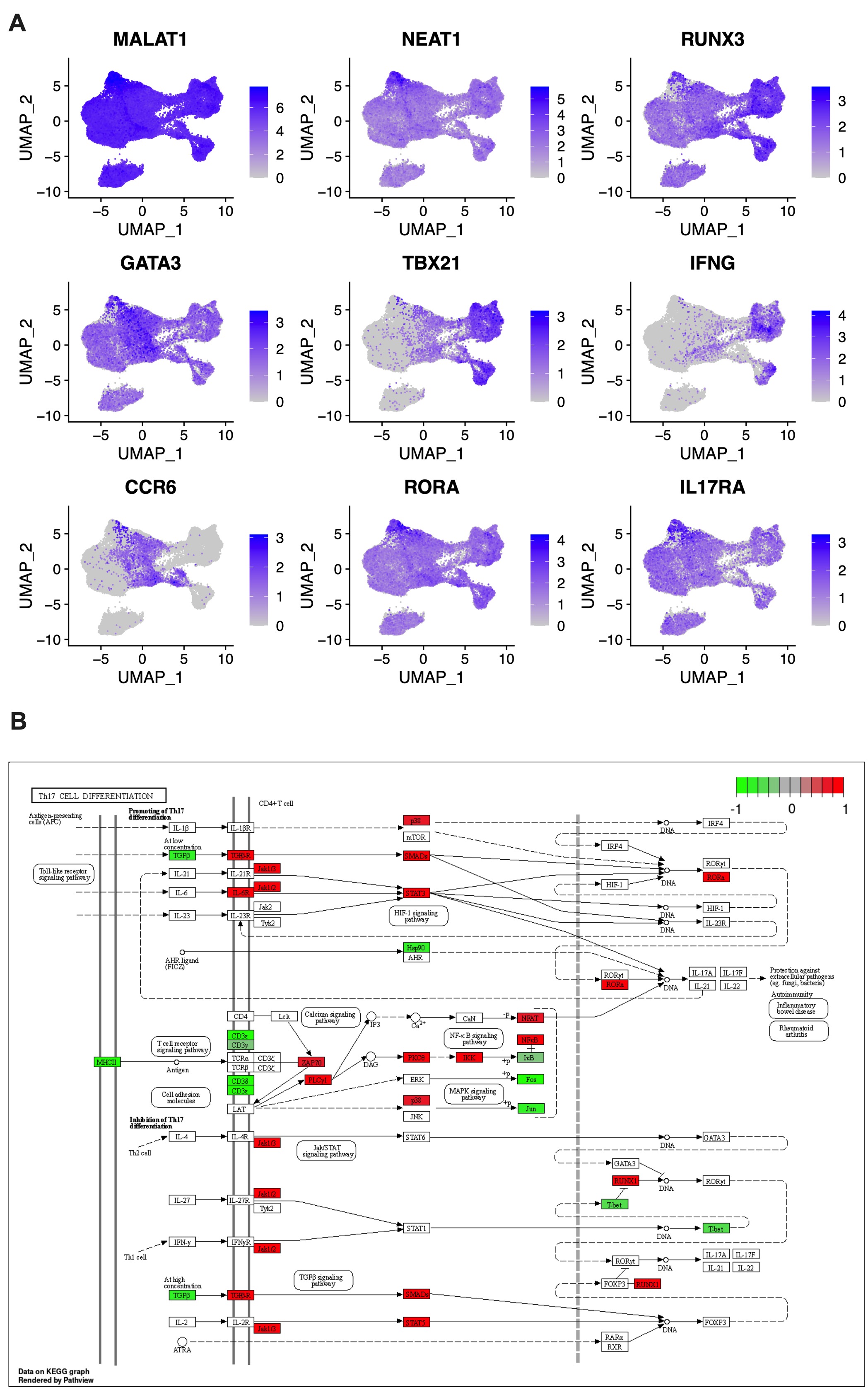

Supplement: Supplementary file 1 [file vaccines-11-01730-s001.zip › vaccines-2662602-supplementary/Supplementary Files/FigS7.jpg]
